# Effects of Cannabis on Glutamatergic Neurotransmission: The Interplay between Cannabinoids and Glutamate

**DOI:** 10.3390/cells13131130

**Published:** 2024-06-29

**Authors:** Kawsar U. Chowdhury, Madison Elizabeth Holden, Miles T. Wiley, Vishnu Suppiramaniam, Miranda N. Reed

**Affiliations:** 1 Department of Drug Discovery and Development, Auburn University, Auburn, AL 36849, USA; kzc0089@auburn.edu (K.U.C.); mtw0055@auburn.edu (M.T.W.); 2 Department of Molecular and Cellular Biology, College of Science and Mathematics, Kennesaw State University, Kennesaw, GA 30144, USA; 3 College of Science and Mathematics, Auburn University, Auburn, AL 36849, USA; meh0162@auburn.edu; 4 Center for Neuroscience Initiative, Auburn University, Auburn, AL 36849, USA

**Keywords:** cannabinoid, glutamate, synapse, memory, LTP

## Abstract

There has been a significant increase in the consumption of cannabis for both recreational and medicinal purposes in recent years, and its use can have long-term consequences on cognitive functions, including memory. Here, we review the immediate and long-term effects of cannabis and its derivatives on glutamatergic neurotransmission, with a focus on both the presynaptic and postsynaptic alterations. Several factors can influence cannabinoid-mediated changes in glutamatergic neurotransmission, including dosage, sex, age, and frequency of use. Acute exposure to cannabis typically inhibits glutamate release, whereas chronic use tends to increase glutamate release. Conversely, the postsynaptic alterations are more complicated than the presynaptic effects, as cannabis can affect the glutamate receptor expression and the downstream signaling of glutamate. All these effects ultimately influence cognitive functions, particularly memory. This review will cover the current research on glutamate–cannabis interactions, as well as the future directions of research needed to understand cannabis-related health effects and neurological and psychological aspects of cannabis use.

## 1. Introduction

According to US News, twenty-four states in the United States have legalized the use of cannabis [1]. Despite this, cannabis remains the most used federally illegal drug in the United States, with approximately 48.2 million people reported to have used cannabis at least once in 2019 [2]. During the Covid-19 pandemic of 2021, over 200 million people used cannabis-derived drugs worldwide [3]. Notably, its use has also increased among pregnant women despite evidence that it crosses the placental barrier [4]. Between 2002–2003 and 2016–2017, the use of cannabis increased from 3.4% to 7.0% among pregnant women [5]. A recent study revealed that following the legalization of cannabis in Colorado, there was a 69% increase in neonatal exposure to cannabis from the policy change in 2012 to 2014 [6]. Given this increase in use, the long-term consequences of cannabis must be further explored.

The use of cannabis is not limited to recreational purposes but is also for medicinal reasons, including treating or preventing vomiting, chronic pain, and seizures [7]. Cannabis exerts its effect primarily through two kinds of G-protein-coupled receptors: cannabinoid 1 (CB_1_) and cannabinoid 2 (CB_2_) receptors [8]. The analysis of CB_1_ receptor distribution in humans, as well as in rodents, indicates that CB_1_ receptors are primarily expressed in the central nervous system (CNS) [9,10,11,12], predominantly in the cortex, hippocampus, amygdala, basal ganglia, and cerebellum, with minimal expression in the peripheral nervous system [13,14]. The distribution of CB_2_ receptors is quite different from that of CB_1_ receptors, with the former being predominantly expressed in the periphery. CB_2_ receptors are expressed in the immunomodulatory organs of the body, like the spleen, tonsils, B cells, monocytes, and T-cells [15,16], and are responsible for controlling inflammation [17]. To some extent, CB_2_ receptors are also expressed in the neurons, where they help to modulate the same immunomodulatory actions of injured or dying cells [18,19].

Endocannabinoids, the endogenous agonists for cannabinoid receptors, include N-arachidonoyl ethanolamine (AEA) and 2-arachidonylglycerol (2-AG) [20], the most prevalent regulators of synaptic function. Endocannabinoids influence the endogenous effects of cannabis receptors and act to control neurotransmission, or the transfer of information from one neuron to another, with the help of neurotransmitters. Glutamate is an excitatory neurotransmitter and plays a crucial role in memory formation [21]. This review provides an overview of the effects of endocannabinoids versus exogenous cannabinoids on glutamatergic neurotransmission. We will discuss the influences of both acute and chronic cannabinoid exposure on glutamatergic neurotransmission. In addition, we will explore the mechanisms by which cannabis and its derivatives disrupt glutamatergic neurotransmission, with a focus on alterations occurring in both pre- and postsynaptic functions. When available, we will also discuss the distinction between alterations observed following prenatal versus adult exposure to cannabis. To identify relevant articles for this review, a comprehensive search was conducted using the following keywords: “Cannabis”, “cannabinoid”, “cannabidiol”, “THC”, “tetrahydrocannabinol”, “cannabis receptor”, “cannabis agonist”, “CB_1_ agonist”, and its effect on “glutamatergic neurotransmission”, “glutamate”, and terms related to the specific proteins discussed in this review.

## 2. Endocannabinoid–Glutamate Interplay

Neurotransmission, the transfer of information through the medium of chemicals, occurs through synapses, the junctions between neurons, namely the presynaptic and postsynaptic neurons. Glutamate, the major excitatory neurotransmitter in the brain, was recognized as a neurotransmitter for the first time in 1984 [22]. Glutamate is stored within presynaptic vesicles, and three different types of vesicular glutamate transporters (VGLUT1, VGLUT2, and VGLUT3) help in transporting glutamate into synaptic vesicles [23]. Upon stimulation, the vesicles fuse with the presynaptic membrane to release glutamate into the synaptic cleft. The released glutamate then binds to metabotropic receptors and ionotropic receptors [24]. Postsynaptic ionotropic receptors play a significant role in learning and memory and, thus, will be the focus of this review. More information regarding the relationship between endocannabinoids and metabotropic glutamate receptors can be found elsewhere [25,26].

Once glutamate reaches the postsynaptic membrane, it binds to α-amino-3-hydroxy-5-methyl-4-isoxazolepropionic acid (AMPA) receptors or N-methyl D-Aspartate (NMDA) receptor subtypes of inotropic glutamate receptors. This binding activates downstream signaling that contributes to memory formation through activity-dependent changes in synapses, known as synaptic plasticity. Long-term potentiation (LTP) and long-term depression (LTD) are the two components of synaptic plasticity that play a crucial role in memory formation. During LTP, several changes happen, including the activation, formation, and recruitment of new AMPA and NMDA receptors. The induction of LTP requires Ca^2+^ entry, which can occur through NMDA receptors or Ca^2+^ channels. Ca^2+^ entry activates various downstream signaling molecules, including calcium-calmodulin-dependent protein kinase II (CAMKII) [27], protein kinase C, PKA, tyrosine kinase Src, and mitogen-activated protein kinase (MAPK) [28]. For LTP, this signaling requires the synthesis of mRNA, mRNA translation, protein kinase A (PKA), or Cyclic adenosine 3,5-monophosphate (cAMP) [29,30,31]. These signaling pathways converge on different transcription factors that regulate protein synthesis to create memory and potentiate LTP [32]. cAMP response element binding protein (CREB) is a transcription factor that plays a crucial role in LTP [33]. Other molecules, such as ERK [34] and PI3K [35], also play essential roles in LTP.

Endocannabinoids are lipid molecules that can modulate neurotransmission through retrograde signaling [36]. Upon postsynaptic depolarization or receptor activation, endocannabinoids are released from postsynaptic neurons to act on presynaptic CB_1_ receptors that induce a short-term suppression of neurotransmitter release, a process termed endocannabinoid-mediated short-term depression, at both excitatory [37,38] and inhibitory [39,40] synapses. In the case of overexcitation of the neuron by glutamate, Ca^2+^ overload can occur, leading to excitotoxicity and neuronal death [41]. Endocannabinoids play a crucial role by inhibiting glutamate release from the presynaptic neuron [42] and inhibiting the NMDA receptors, resulting in decreased entry of NMDA-induced Ca^2+^ in the postsynaptic neuron [43]. For a full review of the role that endocannabinoids play in synaptic functioning, see [44].

Since cannabinoid receptors and glutamate mediate neurotransmission, they can mutually modulate each other’s functions. Numerous studies have demonstrated that compounds like THC [45], WIN 55-212,2 [46,47,48], and HU-210 [49], which target cannabinoid receptors, can disrupt long-term potentiation (LTP) regardless of exposure timing—whether prenatal [46,47] or postnatal [49]—and regardless of exposure duration, whether acute [48] or chronic [45,46,47,49]. Several studies have investigated the effects of cannabinoids on various aspects of glutamate-mediated neurotransmission These effects can differ based on factors like acute versus chronic exposure, age, sex, timing, and frequency of exposure. However, currently, there is no comprehensive review summarizing all these different effects. Therefore, the goal here is to review the effects of all these factors on glutamatergic neurotransmission and the downstream pathways involved in memory formation. Numerous prior reviews (e.g., [50,51,52,53]) have discussed the biochemical pathways for the synthesis, degradation, and cellular actions of endogenous cannabinoids. Here, we focus on the consequences of retrograde signaling on glutamate neurotransmission.

## 3. Effects of Cannabis on Presynaptic Glutamatergic Neurotransmission

Here, we review the impact of cannabis on the presynaptic phase of glutamatergic neurotransmission. As mentioned above, glutamate is transported by different vesicular glutamate transporters (VGLUT1-3). While the acute effects of cannabis on VGLUT have not been examined to our knowledge, chronic use of the cannabis agonist WIN 55,212-2 increases VGLUT1 in the hippocampi of male rats [46]. However, chronic administration of the CB_1_ agonist CP 55,940 does not affect VGLUT3 in the prefrontal cortices of male rats [54]. This discrepant finding may be due to the different brain regions studied, with the former focusing on the hippocampus and the latter on the PFC. Also, VGLUT3 is not expressed in excitatory terminals, unlike VGLUT1 and VGLUT2 [55]. Interestingly, chronic administration of delta-9-THC during adolescence at a dose of 10 mg/kg increases VGLUT1 during adulthood in the medial prefrontal cortex (mPFC) and was associated with schizophrenia-like behaviors in rodents [56], which may help explain why cannabis use is a predisposing or risk factor for schizophrenia in humans [57,58]. While VGLUT1 helps transport glutamate, the release of glutamate within the synaptic cleft depends on calcium.

While chronic exposure of cannabis on calcium channels has not been examined to our knowledge, acute exposure to cannabinoid compounds inhibited all three different types of calcium channels [59]. Since calcium controls the fusion of the synaptic vesicle with the synaptic membrane, its inhibition can limit the fusion process and, subsequently, the release of neurotransmitters. Additionally, acute treatment of cerebellar granule cells with CB_1_ agonist HU-210 at a dose of 5 μM was shown to reduce the number of synaptic vesicles in the active zone, leading to a decreased supply of neurotransmitters [60]. This inhibition was attributed to the inhibition of cAMP, as cAMP inhibition inhibits RIM1α, the active zone protein that modulates priming of the synaptic vesicle in the active zone. Similar results were also found when cerebellar granule cells were treated acutely with the CB_1_ agonist Hu-210. The inhibition was attributed to the inhibition of adenylyl cyclase, leading to the inhibition of the cAMP/EPAC/PLC pathway and eventually inhibiting the activity of active zone protein Munc-13 [61]. All these factors lead to decreased neurotransmitter release from the synaptic vesicles following acute treatment.

In both human cell cultures and mouse hippocampal slices, it was found that acute treatment with the CB_1_ agonist WIN 55,212- mesylate caused Gβ/γ-mediated inhibition of Ca^2+^ channels, which decreased the vesicular release of the neurotransmitter and also inhibited adenylyl cyclase, resulting in a decrease in cAMP levels [62]. However, through electron microscopy, the researchers found a surprising result: inhibition of cAMP, instead of suppressing synaptic vesicles, caused an increased supply of synaptic vesicles from the readily released pools in the active zone by PKA-mediated phosphorylation of synapsin, leading to increased release of glutamate induced by high-frequency action potentials. However, this experiment was conducted in cultured neurons and, in a physiological context, the CB_1_ receptor is not easily able to enhance glutamate release because the inhibition of cAMP also suppresses the key active zone proteins, like Munc-13 and RIMα, necessary for vesicle docking. Additionally, cannabis-induced inhibition of calcium release exacerbates the hindrance to neurotransmitter release. These insights shed light on the intricate mechanisms underlying cannabinoid actions on the release mechanisms of glutamate presynaptically.

The fusion and exocytosis of the synaptic vesicle are controlled by synaptophysin, the vesicular protein. Acute treatment of cortical neuronal cell culture with a low concentration of THC (10 nM) increased the expression of synaptophysin [63], aligning with the result of [62], which showed the increased release and supply of synaptic vesicles in the active zone. However, the treatment of the cell with a high concentration of THC (1 μM) decreased the expression of synaptophysin due to neurotoxicity. On the other hand, chronic treatment of adolescent male rats with the CB_1_ agonist CP 55,940 caused no change in synaptophysin expression in the hippocampus or PFC [54], but when female adolescent rats were treated with THC, chronic treatment decreased the expression of synaptophysin in their PFCs as adult rats but not in their hippocampi [64]. In comparing these two studies, [54] focused on male rats exclusively, while [64] solely examined female rats. This divergence in sexes could potentially explain why the vesicular protein synaptophysin remained unchanged in males but decreased in females. [54] did not explore postsynaptic markers, but it is worth noting that in some instances, compensatory mechanisms may lead to unchanged presynaptic structures while the postsynaptic ones are altered. Additionally, we have corroborated this observation, demonstrating that synaptophysin, a presynaptic protein, remained unaltered, while postsynaptic markers exhibited changes following prenatal exposure to WIN 55,212-2 [46], a topic to be elaborated further in a subsequent section of this review.

To summarize, the impact of cannabinoid compounds on the presynaptic phase of glutamatergic neurotransmission is complex and is summarized in Table 1. Acute administration of cannabinoid compounds primarily causes the inhibition of glutamate release by targeting various components of the release machinery, including calcium channels, RIMα, Munc-13, and synapsin. In contrast, chronic exposure can increase glutamate release and is associated with psychological problems, including schizophrenia-like behavior. Overall, the effect of cannabinoids on presynaptic glutamate neurotransmission is complex and depends on factors like treatment timing, dosage, and age. Future studies are needed to fully elucidate the long-term effects of cannabis exposure on neurological as well as psychological conditions.

## 4. Effects of Cannabis on Postsynaptic Glutamatergic Neurotransmission

### 4.1. Effects of Cannabis on Glutamate Receptors

After glutamate is released into the synaptic cleft, it binds to receptors on the postsynaptic surface. These receptors are located in an area known as the postsynaptic density (PSD). PSD95 acts as a scaffolding protein and holds the postsynaptic receptors in position. While acute exposure has been shown to increase PSD95 in the PFC [65], chronic exposure decreased PSD95 in the PFC [64]. In contrast, neither acute nor chronic exposure altered PSD95 in the hippocampus [64,65]. These findings highlight the region-specific effects of cannabis exposure on synaptic proteins and suggest that the PFC is more susceptible to changes in PSD95 levels compared to the hippocampus.

Once glutamate is released and reaches the postsynaptic surface, it binds to AMPA receptors, leading to depolarization of the neuron and subsequent activation of NMDA channels. Researchers have also investigated the effects of cannabinoid compounds on the expression of AMPA and NMDA subunits. Chronic prenatal exposure to cannabinoid compounds alters the differentiation of glutamatergic neurons in the cerebral cortex [66], which will eventually influence the expression of glutamatergic neurons and receptors. Likewise, chronic prenatal and perinatal treatment with 5 mg/Kg of THC decreases the expression of GluA1 and GluA2/A3 in the cerebellum when examined at three different postnatal days (PND 20, PND 30, and PND 70), indicating that prenatal exposure may induce permanent alterations in AMPA receptor expression following prenatal exposure to cannabinoids [67]. However, when administered during adolescence to female rats, chronic exposure to THC at a high dose (twice a day throughout the whole treatment paradigm: PND 35–PND 37, 2.5 mg/kg; PND 35–PND 37, 5 mg/kg; and PND 38–PND 41, 10 mg/kg) caused increased expression of GluA1 in the adult PFC but no change in GluA2 [68]. Nevertheless, the same treatment protocol during adolescence showed increased expression of GluA1 and GluA2, both by about 80% and 300%, respectively, in the hippocampi of male rats but no change in their PFCs [69]. In addition, our study also revealed comparable outcomes in the hippocampi of male rats treated with WIN 55,212-2 at a dosage of 2 mg/kg [46], notably, decreases in GluA1 levels. In a separate study, in which both male and female mice were treated chronically for seven consecutive days with a moderate dose of THC (10 mg/kg), hippocampal expression of GluA1 was decreased without any change in GluA2 expression [70], though they did not separate out the changes based on sex. Together, these studies suggest that cannabinoid exposure can cause long-lasting changes in AMPA, though the brain region primarily affected appears to depend on sex, with males exhibiting dysregulation of AMPA receptors in the hippocampus, while the PFC is affected in females.

NMDA receptors can also be affected by exposure to cannabis. Acute treatment with 0.3 mg/kg of THC decreased the expression of GluN2A, while there was no effect on GluN2B expression in the hippocampi of male rats [71]. In the same experimental setup, when an ultra-low dose of THC (0.002 mg/kg) was administered, there were no changes found in the hippocampus. Chronic treatment of mice with THC for seven consecutive days, however, decreased the expression of GluN2A and GluN2B in the hippocampus, while GluN1 levels remained unchanged [45,70]. Prenatal treatment with the cannabinoid agonist WIN 55, 212-2 also decreased the expression of GluN2A, with no changes in GluN2B expression in the hippocampi of male rats [46]. Moreover, when treated with a heavy dose of THC (twice a day throughout the entire course of treatment; PND 35–PND 37, 2.5 mg/kg; PND 35–PND 37, 5 mg/kg; and PND 38–PND 41, 10 mg/kg) during adolescence, both males and females exhibited increased expression of GluN2B during adulthood, even two months after the cessation of treatment. However, GluN2A levels remained unchanged [68,69]. According to the natural maturation process, GluN2B is expressed more during development and early ages, but molecular switching happens during adulthood, leading to decreased GluN2B expression compared to GluN2A [72]. THC caused dysregulation in this molecular switching. When the gene expression was examined, in vitro treatment of the human stem cell-derived neurons showed decreased expression of *GRIA1, GRIA2, GRIN2A,* and *GRIN2B* genes [73].

In exploring the mechanistic details of these alterations, research has shown that exposure to THC is linked to altered DNA methylation patterns in offspring, which eventually affect the genes involved in glutamatergic neurotransmission [74,75]. The *Dlg4* gene, which encodes the scaffolding protein PSD-95, exhibits epigenetic dysregulation after THC exposure, resulting in abnormal glutamatergic transmission [74]. Another study identified that cannabis use leads to differential methylation of genes, specifically affecting the pathways that are related to glutamatergic neurotransmission and long-term potentiation [76]. The preceding analysis of cannabinoid effects on AMPA and NMDA receptor subunits reveals a complex interplay between prenatal, perinatal, and adolescent exposure and subsequent alterations in receptor expression across brain regions. These discrepancies in the effects may stem from several factors, including differences in experimental protocols, animal models, dosages, treatment durations, and the brain regions studied.

### 4.2. Effect of Cannabis on Downstream Signaling of Glutamate Receptors

The downstream signaling of glutamate receptors that influence learning and memory can also be affected by cannabis exposure. Earlier, we discussed that once glutamate binds to the AMPA receptors, the postsynaptic membrane is depolarized, followed by the activation of NMDA channels. Only then do NMDA channels allow the entry of Ca^2+^ ions, which ultimately activate the second messenger system to aid in the formation of memory. Some other sources also supply Ca^2+^, like the endoplasmic reticulum. The endoplasmic reticulum releases calcium through the ryanodine receptor-sensitive calcium channel to increase the supply of intracellular calcium in the post-synaptic neuron. Chronic activation of cannabis receptors by CB_1_ agonist WIN 55,212-2 decreased the supply of intracellular calcium, resulting in the alteration of the second messenger system’s activity [77]. However, when treated acutely, the cannabinoid agonist HU-210 increased intracellular calcium [78]. Following calcium entry, a kinase enzyme called protein Kinase C (PKC) is activated by calcium. During the long-term storage of memory and LTP, PKC-dependent phosphorylation of NMDARs causes the activation of NMDARs and helps to express more AMPARs in the synaptic surface [79]. PKC has several neuron-specific substrates required for learning and the formation of memories [80], among which neurogranin is one of the proteins located post-synaptically. Neurogranin modulates the binding and unbinding of the calcium-binding protein calmodulin (CaM), which controls calcium binding and leads to memory formation [81,82]. Acutely activated CB_1_ receptors stimulate the phosphorylation of PKC and the phosphorylation of neurogranin, leading to no availability of free CaM for binding with Ca^2+^ to help in memory formation. This is one of the proposed mechanisms for how the acute activation of CB_1_ interferes with short-term memory formation [83]. Likewise, protein kinase A (PKA) is inhibited by acute exposure to CB_1_ agonists in cell culture [84]. Thus, chronic cannabis exposure can affect memory by decreasing intracellular calcium, which eventually affects the second messenger system crucial for memory formation. Conversely, acute exposure induces transient alterations that may initially modulate synaptic plasticity but ultimately interfere with memory consolidation processes. This highlights the intricate interplay between cannabinoid receptor activation, calcium signaling, and kinase activity in shaping the effects of cannabis on memory function.

Central to the downstream signaling of NMDARs are the Mitogen-activated protein (MAP) kinases [85]. The three most important MAP kinases signaling in the central and peripheral nervous systems are the extracellular signal-related kinases (ERKs), p38 MAP kinases, and C-Jun N-terminal kinases [85,86]. It was already established from prior research that endogenous cannabinoids activate the MAP kinase signal transduction pathway [87]. Transfection of Chinese hamster ovary (CHO) cells with CB_1_ receptors, followed by treatment of these cells with three CB_1_ agonists, CP 55,940, THC, and WIN 55,212-2, revealed that all activated the MAP kinases [88]. In addition to that, the agonists showed dose dependency while activating ERK1/2, and the relative ranking of the three different agonists was as follows: CP 55,940 > THC > WIN 55,212-2 [88]. THC also activated the MAPK/ERK in the dorsal striatum and the nucleus accumbens of treated mice [89]. In human astrocytoma cells, acute exposure to the CB_1_ agonist HU-210 induced ERK activation, which was the downstream consequence of Gi dissociation and PI3K/PKB activation [90]. Several other research labs showed, using different cell lines, that PI3K/PKB is needed to activate ERK. Apart from that, it was also shown that inhibition of adenylyl cyclase, followed by the inhibition of PKA, is one of the predominant pathways for ERK activation due to CB_1_ stimulation [84,91,92,93]. In the hippocampus, THC also activated ERK [94]. But, in the hippocampus, the ERK activation was not PI3K/PKB dependent. Instead, it was dependent on cAMP. Also, THC induced the expression of several genes (c-Fos protein, *Zif268*, and BDNF mRNAs) following the activation of ERK [94]. In the caudate putamen and cerebella of the rats, acute exposure to THC increased ERK even though there was no change in the hippocampus, nucleus accumbens, and prefrontal cortex with the same acute dose. However, when the same animal was exposed chronically, homeostatic adaptation led to no ERK change in the caudate putamen and cerebellum but significant activation in the hippocampus and cerebellum. Also, the role of Ras-Grf1 in this dose-dependent ERK activation and adaptation was found, as there was no effect on ERK when Ras-grf1 knockout mice were used [95]. In the cerebral frontal cortex, acute exposure to the CB_1_ agonist WIN increased c-raf, ERK, MEK, and pERK, yet, when administered chronically for five consecutive days, there was no effect due to desensitization of the CB_1_ receptors [96]. However, in cultured neuronal stem cells derived from mice, the CB_1_ agonist arachidonoyl-2-chloroethyl amide (ACEA) promotes neuronal maturation and differentiation by inhibiting the ERK1/2 pathway [97]. Acute exposure to the CB_1_ agonist cannabidiol at two different concentrations (5 μm and 10 μM) phosphorylated Erk ½, though the phosphorylation was higher at 5 μm than 10 μm [93]. These different effects in different models (in vitro and in vivo) suggest that the cannabinoid-induced effect of ERK acts differently in various models and treatments.

In summary, it appears that the relationship between the cannabinoid receptors and the MAP kinase signaling pathway is complicated. While both endogenous cannabinoids and various CB_1_ agonists consistently activate MAP kinases, the specific mechanisms vary across different cell types, brain regions, and experimental conditions. These cannabinoids exhibit diverse effects on ERK activation, from promoting neuronal maturation and differentiation to modulating gene expression and adaptation, both in vitro and in vivo. All these diverse effects underscore the importance of considering the specific context and conditions when studying cannabinoid-mediated signaling, highlighting the need for further research to elucidate the precise mechanisms and therapeutic implications of cannabinoid-induced ERK activation.

Apart from ERK, another essential part of the MAPK signaling pathway is p38 MAPK. In the hippocampus, when LTP is induced, p38 MAPK is activated [98], while inhibition of p38 MAPK protects the neuron from over-excitation from glutamatergic neurotransmission [99]. In terms of the effect of CB_1_ receptors on p38 MAPK, incubation of both rat and mouse hippocampi with exogenous as well as endogenous CB_1_ receptor agonists has been shown to increase the phosphorylation of p38 MAPK [100]. A brief exposure (5 min) of CHO cells to 1 μM of THC activated p38 and JNK activity [101], whereas prolonged exposure (6 h) of AF5 cell lines to 3 μM of THC led to inhibition of p38 MAPK activation [102]. Cannabidiol was also found to inhibit p38 when examined in PC12 cells cultured for 36 h with cannabidiol at 10^−6^ to 10^−4^ M concentrations [103]. Treatment of mice with THC for seven consecutive days caused phosphorylation of p38 MAPK [45]. Numerous factors, from variations in cell types to the utilization of animal models, cannabinoid exposure duration, and concentrations, coupled with the distinct pharmacological properties of THC and cannabidiol, contribute to the observed differences in these responses. Thus, in summary, p38 MAPK and phosphorylation of p38 play a crucial role in glutamatergic neurotransmission, particularly in synaptic plasticity mechanisms like LTP, as inhibition of p38 can protect against over-excitation caused by glutamatergic neurotransmission. While in most cases, CB_1_ receptor agonists induce phosphorylation of p38 MAPK in rat and mice hippocampi, THC exhibits varied effects on p38 activation, stimulating it in CHO cells but inhibiting it in the AF5 cell line, while cannabidiol suppresses p38 MAPK in PC12 cells. Overall, the involvement of p38 MAPK in glutamatergic neurotransmission and synaptic plasticity adds another layer of complexity to the interaction between cannabinoids and the MAP kinase signaling pathway. These findings highlight the intricate interplay between cannabinoids and p38 MAPK signaling, suggesting that the effects may be context dependent. Further research is needed to elucidate the precise mechanisms underlying these discrepancies and their potential implications for synaptic plasticity and neuronal function.

To summarize, the MAPK signaling pathway, particularly ERK and p38 MAPK, is integral to various neuronal processes, including synaptic plasticity and neurotransmitter regulation. The relationship between cannabinoids and MAP kinase signaling is complex and context-dependent, influenced by factors such as cell type, brain region, and treatment duration. Understanding these intricacies is essential for elucidating the therapeutic potential of cannabinoid-mediated signaling pathways and their implications for neurological disorders.

Another downstream signaling pathway of NMDA receptors is PI3K/Akt, and it plays neuroprotective roles against neurotoxicity [104,105]. Acute activation of the CB_1_ receptor with THC has been found to activate the PI3K/Akt pathway, provide protection against neurotoxicity, and help in neuronal survival [106,107]. In this activation process, the CB_1_ agonist THC phosphorylates Akt, which depends on a PI3K-dependent pathway and protects the neuron from external toxic stimuli like glutamate-mediated neurotoxicity. The downstream signaling target of PI3K/AKT is mTOR, which is a serine/threonine kinase, also called the mammalian target of rapamycin [108]. mTOR plays a crucial role in developing neurons and modifying synaptic strength [109]. Acute treatment of mice with THC phosphorylated mTOR and led to increased protein synthesis [110]. The increased synthesis of protein was confirmed using the protein synthesis inhibitor anisomycin. The increased protein synthesis was found to correlate with the amnesic-like effects of THC. When anisomycin was used, it improved the amnesic-like effects of THC. Also, the pharmacological blockade of mTOR improved the amnesic-like effects [111].

As all the pathways converge at the CREB pathway, the machinery that helps in the new protein synthesis for memory formation [112], the effect of the CB_1_ agonist on the activity of CREB was also assessed. Two separate studies on male rats revealed that acute treatment with THC, at doses ranging from 2.5 to 10 mg/kg in one study [113] and at 15 mg/kg in another [95], increased CREB phosphorylation (pCREB) in the hippocampus, caudate putamen, and cerebellum [95,113]. However, the response to chronic THC exposure revealed a different pattern. Chronic treatment resulted in adaptations within the same brain regions, attenuating the changes seen during acute exposure. Interestingly, chronic THC exposure at a dose of 15 mg/kg activated CREB in the prefrontal cortex (PFC) only. Rubino et al. did not observe any deviations in cerebellar activity, contrasting with Casu et al.′s discovery of diminished pCREB expression in the same area post-prolonged THC exposure at 10 mg/kg [113]. Both investigations were limited to male rats. The contradiction in results could be attributed to several factors. Notably, Rubino’s protocol entailed a higher THC dosage (15 mg/kg) administered bi-daily for 6.5 days. In contrast, Casu’s regimen utilized a marginally lower dose (10 mg/kg), which was given once daily over four consecutive weeks. Moreover, chronic THC exposure at 10 mg/kg in mice reduced the expression of both total CREB and pCREB in the hippocampus [70], though they did not separate out the changes based on sex. The differences in the strain and species of the animals (rat vs. mice), as well as the dosage and duration of exposure, may have played a role in such discrepancies.

**Figure 1:**
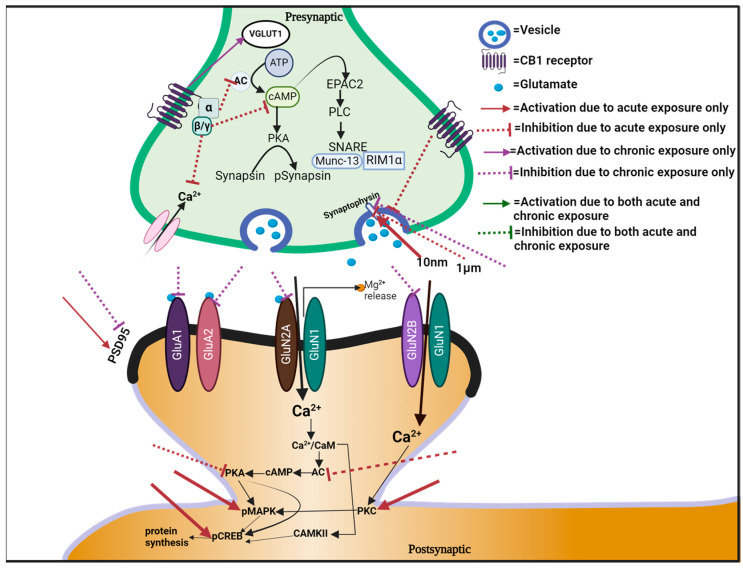
Pictorial illustration of effects of cannabis on glutamatergic neurotransmission.

In summary, the impact of cannabinoid compounds on glutamatergic neurotransmission is intricate, as outlined in Figure 1 and Table 2. This review highlights that both acute and chronic exposure to cannabinoid compounds, particularly THC, can elicit a range of effects on glutamatergic signaling, which may, at times, elicit opposing effects. While acute exposure predominantly exhibits inhibitory effects, chronic exposure can lead to adaptive changes. Furthermore, these effects are contingent upon factors such as brain region, age, sex, and dosage regimen. To fully grasp the mechanisms by which cannabinoids influence glutamatergic neurotransmission in specific brain areas, further research is imperative (Table 3).

## 5. Consequences of Cannabinoid-Mediated Alterations in Glutamatergic Signaling

Changes in glutamate receptor expression and the downstream signaling pathways of these receptors significantly impact cognitive functions such as learning and memory. AMPA and NMDA receptors are crucial in these processes. AMPARs mediate most fast excitatory synaptic transmission, which is critical for synaptic plasticity, with GluA1 and GluA2 subunits playing pivotal roles [114,115,116]. Similarly, NMDARs are essential for learning and memory [117,118,119]. We already discussed that cannabis exposure decreases the expression of GluA1 and GluA2 receptors, which can impair learning and memory. The same theory applies to NMDARs, as they are critical for the induction of long-term potentiation (LTP). Conversely, increased glutamate receptor expression due to cannabis exposure can lead to excitotoxicity due to Ca^2+^ overload [120,121]. Research has shown that chronic exposure to THC activates microglia and astrocytes, producing inflammatory cytokines that lead to neuroinflammation [122]. Chronic exposure to WIN 55,212-2 has also been shown to increase glutamate release, eventually causing excitotoxicity and neurodegeneration [46]. Additionally, the downstream signaling pathways of glutamate receptors, including cAMP, AC, MAPK, and CaMKII, converge on CREB (cAMP response element-binding protein), a key factor in new protein synthesis required for memory formation [112]. Cannabis alters these pathways, thereby affecting protein synthesis and, ultimately, learning and memory.

These changes in glutamatergic neurotransmission pathways, as well as neuroinflammation, can cause alterations in learning and memory. Earlier, it has been discussed that various cannabis compounds, such as THC [45], WIN 55-212,2, [46,47,48], and HU-210 [49], which target cannabinoid receptors, can disrupt LTP, which is a cellular model of learning and memory. Disruption of LTP refers to the decline in cognition. Our recent publication has further substantiated the alteration of cognition through several behavioral tests. Chronic treatment with WIN 55,212-2 was found to alter hippocampus-dependent contextual fear memory and spatial learning and reference memory [46]. Similarly, adolescent exposure to THC has been found to cause declines in prefrontal cortex-based spatial memory [64]. The biochemical reasons behind these cognitive deficits were previously discussed in the context of presynaptic and postsynaptic changes in glutamatergic neurotransmission due to cannabis use. Overall, cannabis use can cause alterations in glutamatergic neurotransmission, leading to learning and memory deficits.

## 6. Discussion

In this comprehensive review, we delve into the impact of cannabis on glutamatergic neurotransmission, a pivotal system involved in fundamental cognitive processes such as memory. As cannabis and its derivatives gain legal recognition for both recreational and medicinal use globally, understanding their influence on neurotransmitter systems becomes increasingly vital.

Numerous studies have elucidated cannabinoids’ ability to modulate glutamate-mediated neurotransmission [123,124,125,126]. Our objective is to synthesize findings from a spectrum of research, encompassing in vitro and in vivo investigations, to delineate cannabis compounds’ multifaceted effects on both presynaptic and postsynaptic facets of glutamatergic neurotransmission.

This review underscores the nuanced outcomes associated with cannabis exposure, influenced by variables such as dosage, duration of administration, and the specific brain regions under scrutiny. Through a meticulous examination of the existing literature, we offer insights into the intricate interplay between cannabis and glutamatergic neurotransmission, thereby contributing to a deeper comprehension of its broader neurological ramifications.

This review also highlights the differential impact of acute and chronic cannabis exposure on glutamatergic neurotransmission. Acute exposure to CB_1_ agonists inhibits glutamate release through the modulation of calcium channels and synaptic vesicle dynamics. Conversely, chronic exposure leads to adaptations, including altered receptor expression and downstream signaling pathways. These adaptations exhibit variability across brain regions and species, adding complexity to the understanding of cannabinoids’ effects on glutamatergic neurotransmission.

This review explores the modulation of downstream signaling pathways crucial for memory formation by cannabinoids. It elucidates the involvement of ERK, p38 MAPK, PI3K/Akt, and mTOR in mediating cannabis’s effects on cognitive processes. The findings suggest that CB_1_ receptor activation may exert protective or disruptive effects on these signaling pathways, contingent upon the context and duration of exposure.

Cannabinoids can activate CB_1_ receptors on astrocytes, subsequently stimulating glutamate release. This release of glutamate from astrocytes may modulate neuronal activity [127,128,129]. However, due to the limited research on astrocytic glutamate release, we have not included a detailed discussion in our review article. Further investigation in this area is warranted.

In addition to cannabinoids, cannabis contains several other biologically active compounds, including flavonoids, terpenoids, stilbenoids, and alkaloids [130]. While research on the effects of these compounds on glutamatergic neurotransmission is not as extensive as that on cannabinoids, several studies have explored their impact in other contexts [131,132,133,134], and a few have examined the consequences on glutamate. For example, one notable flavonoid, apigenin, has been found to inhibit glutamate release in the hippocampus [135]. Another flavonoid, quercetin, reduces cellular calcium concentration, thereby protecting cells from glutamate excitotoxicity [136]. Terpenoids, another class of biologically active molecules in cannabis, exert neuroprotective effects by modulating the PI3K/Akt pathway, a crucial step in glutamatergic neurotransmission [137]. Similarly, resveratrol, a stilbenoid, has been shown to modulate the PI3K/Akt pathway, resulting in neuroprotection [138]. Furthermore, an alkaloid found in cannabis, harmine, has been demonstrated to increase glutamate clearance from the synapse, providing neuroprotection in cases of glutamate overload [139]. These findings highlight the potential of various biologically active compounds in cannabis to influence glutamatergic neurotransmission and offer neuroprotective benefits.

## 7. Conclusions

In summary, this review offers a comprehensive analysis of the complex interplay between cannabis and glutamatergic neurotransmission. It emphasizes the necessity of accounting for multiple variables in cannabis research, including duration of exposure, regional specificity within the brain, and interspecies variations. These insights enhance our comprehension of the psychological ramifications of cannabis use, emphasizing the imperative for additional investigations to clarify the underlying mechanisms and potential ramifications for both public health and clinical intervention.

## Figures and Tables

**Table 1:** The effects of cannabinoid compounds on the presynaptic phase of glutamate-mediated neurotransmission.

Name of the Proteins	Type of Model	Acute or Chronic	Exposure Type	Drug and Dosage	Dose Regimen	Effect of the Treatment	Brain Area of Interest	Reference
VGLUT1	Male mice	Chronic	Intraperitoneal	∆9-THC; 10 mg/kg	PND 28 to PND 48	Increased	mPFC	[56]
VGLUT1	Male Sprague Dawley rats	Chronic	Subcutaneous	WIN 55,212-2; 2 mg/kg	GD 3 to PND 2	Increased	Hippocampus	[46]
VGLUT3	Male Wistar Han rats	Chronic	Intraperitoneal	CP 55,940; 0.15 mg/kg-7 days, 0.2 mg/kg-7 days, 0.3 mg/kg-7 days	PND 29 to PND 50	No changes	mPFC	[54]
Calcium channels (N- and P/Q-types)	Hippocampal cell culture; cells collected on PND 1-PND 4	Acute	In the culture media	WIN 55,212-2 and WIN 55,212-3; 100 nm WIN	Recording was performed between 2 to 10 days of the experiment,	Inhibition of all three types of channels	Hippocampus	[59]
Calcium channels	Male human pluripotent stem cells	Acute	In the culture media	WIN 55,212-2; 10 μm	Into the recording media, just 5 min before the experiment	Inhibition	Human pluripotent cells	[62]
Synaptic vesicle	Cerebellar cell cultures. Cells collected on PND 7; female Wistar albino rat pups	Acute	In the culture media	HU-210; 5 μm	Recording was performed between 7 and 10 DIV	Decrease the number of synaptic vesicles	Cerebellar cell cultures	[60]
cAMP	Cell culture, cells collected from PND 7; from cerebellar granule cells	Acute	In the cell culture media	HU-210; 5 μm	Experiments were carried out 7–8 DIV	Inhibition	Cerebellar cell cultures	[61]
cAMP	Male human pluripotent stem cell	Acute	In the culture media	WIN 55,212-2; 10 μm	Into the recording media, just 5 min before the experiment	Inhibition	Human pluripotent cells	[62]
RIM1α	Cell culture, cells collected from PND 7; from cerebellar granule cells	Acute	In the cell culture media	HU-210; 5 μm	Experiments were carried out 7–8 DIV	Inhibition	Cerebellar cell cultures	[61]
Munc-13	Cell culture, cells collected from PND 7; from cerebellar granule cells	Acute	In the cell culture media	HU-210; 5 μm	Experiments were carried out 7–8 DIV	Inhibition	Cerebellar cell cultures	[61]
Adenylyl cyclase	Cerebellar cell cultures; Cells collected on PND 7; female Wistar albino rat pups	Acute	In the culture media	HU-210; 5 μm	Recording was performed between 7 and 10 DIV	Decrease the number of synaptic vesicles	Cerebellar cell cultures	[60]
Adenylyl cyclase	Male human pluripotent stem cell	Acute	In the culture media	WIN 55,212-2; 10 μm	Into the recording media, just 5 min before the experiment	Inhibition	Human pluripotent cells	[62]
Synaptophysin	Cortical neuronal cell culture: collected in embryonic day 17 from the ventral part of the diencephalon.	Acute	In the culture media	THC (low concentration-10 nm; High concentration-1 μm)	Experiments were carried out on 6 DIV	Low concentration increased synaptophysin expression; high expression induced neurotoxicity	Cortical neuronal cell culture	[63]
Synaptophysin	Male Wistar Han rats	Chronic	Intraperitoneal	CP 55,940; 0.15 mg/kg-7 days, 0.2 mg/kg-7 days, 0.3 mg/kg-7 days	PND 29 to PND 50	No changes	mPFC	[54]
Synaptophysin	Female Sprague Dawley rats	Chronic	Intraperitoneal	THC; PND 35–37, 2.5 mg/kg; PND 38–41, 5 mg/kg; PND 42–45, 10 mg/Kg	PND 35–PND 45	No changes in the hippocampus, but decreased in the PFC	PFC	[64]
Synaptophysin	Male Sprague Dawley Rats	Chronic	Subcutaneous	WIN 55,212-2; 2 mg/kg	GD3 to PND 2	No change	Hippocampus	[46]

**Table 2:** The effect of cannabinoid compounds on glutamate receptors.

Name of the Proteins	Type of Model	Acute or Chronic	Exposure Type	Drug and Dosage	Dose Regimen	Effect of the Treatment	Brain Area of Interest	Reference
PSD 95	Male Swiss mice, 25–30 g, eight weeks of age, and male Wistar rats	Acute	Intracerebral	Cannabidiol; 7, 10, and 30 mg/kg	One dose only	Acute treatment increased PSD 95	All the changes only in the PFC; no changes in the hippocampus	[65]
PSD 95	Female Sprague Dawley rats	Chronic	Intraperitoneal	THC; PND 35–37, 2.5 mg/kg; PND 38–41. 5 mg/kg; PND 42–45, 10 mg/Kg	PND 35–PND 45	No changes in the hippocampus, but decreased expression in the PFC	PFC	[64]
GluA1 GluA2/3	Male and female rats	Chronic	Oral	THC; 5 mg/kg	GD5 to PND 20	Decreased	Cerebellum	[67]
GluA1, GluA2	Female Sprague Dawley rats	Chronic	Intraperitoneal	THC; twice a day; PND 35–37 at 2.5 mg/kg; PND 38–41 at 5 mg/kg; PND 42–45 at 10 mg/kg	PND 35 to PND 45	GluA1- increased, GluA2- no change	PFC	[68]
GluA1	Male Sprague Dawley rats	Chronic	Subcutaneous	WIN 55,212-2; 2 mg/kg	GD3 to PND 2	Decreased	Hippocampus	[46]
GluA1, GluA2	Male Sprague Dawley rats	Chronic	Injection	THC; twice a day; PND 35–37 at 2.5 mg/kg; PND 38–41 at 5 mg/kg; PND 42–45 at 10 mg/kg	PND 35 to PND 45	GluA1- 80% increased GluA2- 300% increased	Hippocampus	[69]
GluA1, GluA2	C57BL/6 mice	Chronic	Intraperitoneal	THC; 10 mg/kg	Seven consecutive days	GluA1- decreased GluA2- no change	Hippocampus	[70]
GluA1, GluN2A, GluN2B	C57BL/6 mice	Chronic	Injection	THC; 10 mg/kg	7 Consecutive days	GluA1, GluN2A, GluN2B- decreased	Hippocampus	[45]
GluA1, GluN2A, GluN2B	C57BL/6 mice	Chronic	Intraperitoneal	THC; 10 mg/kg	7 Consecutive days	GluA1, GluN2A, GluN2B- decreased	Hippocampus	[70]
GluN2A, GluN2B	Male Sprague Dawley rats	Chronic	Subcutaneous	WIN 55,212-2; 2 mg/kg	GD3 to PND 2	GluN2A- decreased, GluN2B- no change	Hippocampus	[46]
GluN2A, GluN2B	Male Wistar rats	Acute	intraperitoneal	THC; 0.3 mg/kg	One dose	GluN2A- decreased, GluN1A/GluN2B- Altered	Dorsal hippocampus	[71]
GluN2A, GluN2B	Female Sprague Dawley rats	Chronic	Intraperitoneal	THC; twice a day; PND 35–37 at 2.5 mg/kg; PND 38–41 at 5 mg/kg; PND 42–45 at 10 mg/kg	PND 35 to PND 45	GluN2B- increased during adulthood, GluN2A- decreased in adulthood. Altered ratio of GluN2A and GluN2B	PFC	[68]
GluN2A, GluN2B	Male Sprague Dawley rats	Chronic	Injection	THC; twice a day; PND 35–37 at 2.5 mg/kg; PND 38–41 at 5 mg/kg; PND 42–45 at 10 mg/kg	PND 35 to PND 45	GluN2B- increased GluN2A- unchanged	Hippocampus	[69]

**Table 3:** The effect of cannabinoid compounds on downstream signaling of glutamate receptors.

Name of the Proteins	Type of Model	Acute or Chronic	Exposure Type	Drug and Dosage	Dose Regimen	Effect of the Treatment	Brain Area of Interest	Reference
PKC	Male, CD1 mice	Acute	Intraperitoneal	THC; 10 mL/kg		Enhanced phosphorylated PKC	Hippocampus	[83]
p42 and p44 MAPK	CHO cells transfected with CB_1_	Acute	In the culture	CP 55,940 > THC > WIN 55,212-2		Activated MAPK		[88]
ERK	U373 MG human astrocytoma cells	Acute (cells were treated 12 h before the experiment)	In the culture	Delta (8)- tetrahydrocannabinol dimethyl heptyl (HU-210)		Activated ERK, mediated by PI3KIB	Hippocampus	[90]
ERK, c-Fos, BDNF	Male CD-1 mice	Acute	Intraperitoneal	THC		Activated ERK	Hippocampus	[94]
Erk, pCREB, c-Fos, FosB	Male Sprague Dawley rats	Acute, Chronic	Intraperitoneal	THC; Acute- 15 mg/kg; Chronic- 15 mg/kg, twice a day, 6.5 days		Acute- increased ERK, pCREB, c-fos in the caudate putamen and cerebellum. Chronic- increased ERK, pCREB, Fos B in the PFC and hippocampus	Caudate putamen, cerebellum, PFC, hippocampus	[95]
Raf-MEK-ERK	Rats	Acute and Chronic	Intraperitoneal	WIN 55,212-2; Acute treatment- 8 mg/kg; Chronic treatment- 2–8 mg/kg	Acute- one dose Chronic treatment- 5 consecutive days	Acute dose- Increased c-Raf, pERK, MEK Chronic treatment- no change	Cerebral frontal cortex	[96]
P38 MAPK	Sprague Dawley rats	Acute	In the bathing solution	WIN 55,212-2; 100 μm	One dose in the bath solution	Activated p38 MAPK	Hippocampus	[100]
P38 MAPK and JNK	Chinese hamster ovary Cells transfected with CB_1_ receptors	Acute	In the culture media	THC; CP 55,940; HU-210	One dose	Activated p38 MAPK and JNK		[101]
Phospho p38 MAPK	PC12 cells	Acute	In the cellular extract	Cannabidiol; 10^−6^ to 10^−4^ M;	Once	Inhibited the phospho p38 MAPK		[103]
Phopho p38 MAPK	AF5 cells	Acute	In the cell culture media	THC; 3 μm	once	Inhibited the phospho p38 MAPK		[102]
PKB, ERK, p38 MAPK	C57BL/6 mice	Chronic		THC; 10 mg/kg	Seven consecutive days	Phosphorylation of PKB, ERK, and p38 MAPK was detected	Hippocampus	[45]
pCREB	Adult male Sprague Dawley albino rats	Acute and Chronic	Intraperitoneal	THC; acute- 2.5, 5 mg/kg or 10 mg/kg; chronic- 10 mg/kg	For acute- one dose only; for chronic- 4 weeks	Acute treatment- increases pCREB Chronic treatment- Markedly attenuate pCREB	Cerebellum	[113]
